# Retrospective analysis of MR imaging characteristics and demographic characteristics of Tropical Endomyocardial fibrosis in a tertiary care centre in South India

**DOI:** 10.1186/1532-429X-18-S1-P286

**Published:** 2016-01-27

**Authors:** RV Leena

**Affiliations:** grid.11586.3b0000000417678969Radiology, Chrisitian Medical College, Vellore, India

## Background

Cardiac MR is helpful in the diagnosis and prognosis of endomyocardial fibrosis[EMF]. Among the two types of endomyocardial fibrosis, tropical endomyocardial fibrosis is geographically restricted to tropical parts of Africa, Latin America and in the southern districts of India, especially along the coastal region of Kerala. Various toxic and environmental factors were postulated as the etiopathogenesis of tropical endomyocardial fibrosis.

Endomyocardial fibrotic wall thickening of apex and subvalvular regions of one or both ventricles results in restrictive pattern. Typical delayed enhancement pattern is the subendocardial enhancement which is prominent in the apex and extending up to the subvalvular regions

## Methods

This study was done in a tertiary care centre in South India. 7 year retrospective analysis of all the cases of endomyocardial fibrosis, which were diagnosed on MRI were included in the study. Retrospective analysis was done using the search tool option in the RIS-PACS software provided by GE. Demographic characteristics, MRI findings and other associated findings were tabulated.

## Results

A total of 11 cases were found, of which 9 were females and 2 were males. Most of the patients were young [less than 40 years]. The socio economic status showed mixed pattern, some (~2) from higher socioeconomic status and others from low to middle socioeconomic status. Place of living also was distributed between various states in Indian subcontinent, and didn't pertain to any costal belt. None of the patients had eosinophilia.

Among the MRI findings, 3 patients had only left ventricular (LV) involvement, 3 patients had only right ventricular (RV) involvement, rest had biventricular involvement with RV involvement more than left ventricle involvement in 4 of them. Subendocardial enhancement was present in all. Two patients had associated mid myocardial enhancement in same or different chambers, hence a possibility of associated hypertrophic cardiomyopathy was also raised. Hypokinesia was present in the involved areas in 8 of the patients. Pulmonary arterial hypertension was seen in 5 patients. Thrombus was present in 2 patients. Associated cardiac cirrhosis was noted in 4 patients. One patient had tuberculous constrictive pericarditis and had underwent pericardial stripping . Presence of calcification was identified in 2 patients, who had additional CT sections. Most of the patients had normal LV systolic function. All the patients underwent medical management.

## Conclusions

1. Apical obliteration with subendocardial enhancement is typical MRI finding

2. MRI findings can mimic Ebstine's anomaly, however subendocardial enhancement will give the diagnostic clue to EMF.

3. Presence of thrombus due to relative ventricular stasis is also common in EMF.

4. Absence of eosinophilia in the tropical EMF. Postulation of combination of factors leading to EMF may be hypothesized in our case series in view of variation in place of living among patients.

**Table 1:** Table 1

Sl No	Place of living	Age	sex	Presence of eosinophilia	Chambers involved	Chambers dilated	Enhancement pattern	Regional wall motion Abnormality	Presence of Pulmonary arterial hypertension	Ejection fraction[LV]	Calcification present	Associated Abnormalities
1	Tamilnadu[TN]	43	M	No	LV	LA, LV	subendocardial	Present	Absent	normal	yes	nil
2	Andhrapradhesh[AP]	34	F	No	LV	LV	subendocardial	Present	Absent	normal	no	nil
3	TN	62	F	No	RV>LV	RA	subendocardial	Present	Present	normal	no	thrombus in RV, cardiac cirrhosis
4	West Bengal	40	F	No	LV	RA, LA	subendocardial, midmyocardial	Absent	Present	normal	no	cardiac cirrhosis
5	TN	27	F	No	RV>LV	RA	subendocardial	Absent	Absent	normal	yes	thrombus in RA, LV,cardiac cirrhosis
6	Bihar	43	F	No	RV>LV	RA	subendocardial, midmyocardial	Present	Absent	normal	no	nil
7	Pondicherry	51	F	No	RV>LV	RA	subendocardial	Present	Absent	reduced	no	nil
8	Bihar	30	F	No	RV	RA	subendocardial	Absent	Absent	normal	no	cardiac cirrhosis
9	AP	23	M	No	LV>RV	RA	subendocardial	Absent	Present	normal	no	Tuberculous constrictive pericarditis
10	Jharkhand	32	F	No	RV	RA	subendocardial	Absent	Present	normal	no	nil
11	TN	61	F	No	RV	RA	subendocardial	Present	Present	normal	no	nil

**Figure 1:**
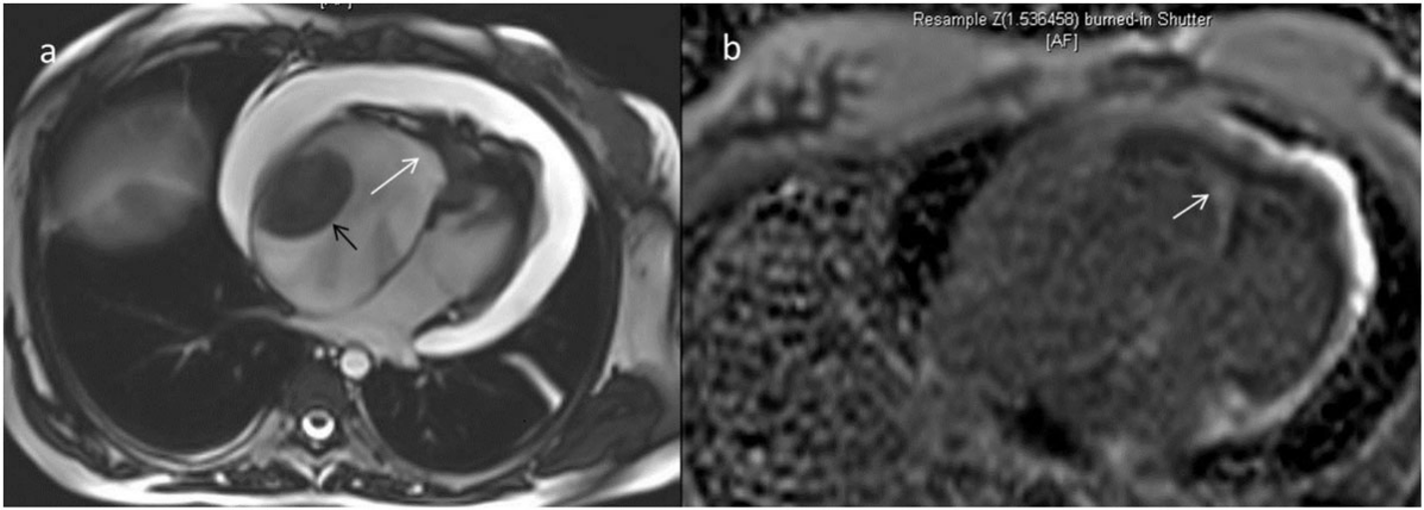
***a) Four chamber view shows RV apex obliteration due to wall thickening [white arrow] and presence of thrombus[black arrow] in dilated right atrium b) Post gadolinium PSIR image shows subendocardial enhancement [white arrow]***.

